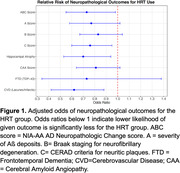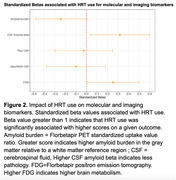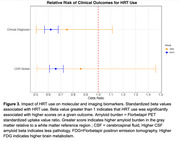# Evidence for a protective effect of hormone replacement therapy on Alzheimer’s disease neuropathological and clinical outcomes across cohorts

**DOI:** 10.1002/alz.092711

**Published:** 2025-01-03

**Authors:** Jennifer Bruno, Jacob Shaw, Victor W. Henderson, Hadi Hosseini

**Affiliations:** ^1^ Stanford, Palo Alto, CA USA; ^2^ John’s Hopkins University School of Medicine, Baltimore, MD USA; ^3^ Stanford University School of Medicine, Stanford, CA USA; ^4^ Stanford University, Stanford, CA USA

## Abstract

**Background:**

More than 2/3 of Alzheimer’s Disease (AD) patients are women, which has led to increased interest in the neurophysiological impact of estrogen decline during menopause. While early evidence suggested that hormone replacement therapy (HRT) may be protective against dementia, more recent studies have found inconclusive or even harmful effects.

**Method:**

We tested the association between HRT use (estrogen or estrogen + progestin) and AD‐related neuropathological outcomes measured on autopsy data in females from the National Alzheimer’s Coordinating Center (NACC) (N = 3423). We also sought to test the association between HRT use and molecular imaging and fluid biomarker measures of AD‐related neuropathology measured in‐vivo in females from the Alzheimer’s Disease Neuroimaging Initiative (ADNI) dataset (N = 2422). Finally, we tested the association between HRT use and clinical outcomes, including Clinical Dementia Rating (CDR) and memory performance, in both ADNI (N = 2422) and NACC (N = 20151) datasets.

**Result:**

We found evidence for a protective effect of HRT on the NIA‐AA AD Neuropathologic Change score (ABC score), hippocampal atrophy, and cerebrovascular disease. We also found evidence for a protective effect of HRT on amyloid burden measured via positron emission tomography (PET) and via cerebrospinal fluid as well as on brain metabolism measured via fluorodeoxyglucose PET. Finally, we found evidence for a protective effect of HRT on dementia diagnosis, CDR and memory performance. All results were significant after controlling for age, APOE genotype and educational level.

**Conclusion:**

We found evidence for small but significant protective effects of HRT across multiple pathological and clinical measures in two different cohorts. First, the protective effect of HRT on neuropathology measured post‐mortem indicates that HRT use is associated with a lower risk of pathology, relative to no HRT use, on this most definitive and final outcome. Second, the protective effect of HRT extended to amyloid biomarkers measured in vivo indicating that future work can utilize these biomarkers to better understand the protective mechanism by which HRT may act. Finally, the protective effect extended beyond pathology to more subtle cognitive outcomes. Together, these results lend understanding to the effects of HRT (or lack thereof) on the aging brain.